# Information Source–Message Effect on Consumers’ Intentions to Adhere to Food Safety Practices When Handling and Preparing Raw Chicken

**DOI:** 10.3390/foods15183170

**Published:** 2026-09-08

**Authors:** Julysa Abril Benitez, Gary Wingenbach, Holli Leggette, Rafael Landaverde, Alejandro Castillo

**Affiliations:** 1 Department of Agricultural Leadership, Education, and Communications, Texas A&M University, College Station, TX 77843, USA; gary.wingenbach@ag.tamu.edu (G.W.); hollileggette@tamu.edu (H.L.); rafael.q@ag.tamu.edu (R.L.); 2 Department of Food Science and Technology, Texas A&M University, College Station, TX 77843, USA; a-castillo@tamu.edu

**Keywords:** communication, experts, foodborne illness, misinformation, social media influencers

## Abstract

Ideal food safety communication provides timely evidence-based information for consumers to protect themselves from foodborne illnesses. We investigated if information sources (experts providing factual information vs. social media influencers (SMIs) providing misinformation), cognitive, affective, and sociodemographic factors influenced participants’ intentions to adhere to food safety practices for raw chicken, a high-risk food. In a quasi-experimental, cross-sectional survey, 727 U.S. adults watched videos about raw chicken food safety from an expert, SMI, or a control. Structural model A explained 56.6% of the variance in intentions. Compared to the control, expert and SMI sources had direct, negative effects on intentions. Experts were associated with greater perceived threat for consuming raw chicken compared to SMI and control videos. Perceived threat and efficacy had significant indirect effects on intentions. Structural model B explained 47.6% of the variance in intentions. Knowledge, trust in science, trust in information source, counterarguing, and sociodemographic factors were insignificant moderators. Food safety communicators should provide clear information about the risks of consuming raw or undercooked chicken and actionable guidance for consumers to avoid foodborne illnesses. Audience segmentation analysis may be an important next step to identify consumer groups and tailor food safety messages to specific consumer needs.

## 1. Introduction

Foodborne illness remains a significant public health issue in the U.S., affecting an estimated 48 million people each year [1]. Between 2014 and 2022, the Centers for Disease Control and Prevention outbreak investigations estimated that 16.5% of foodborne illnesses occurred in home settings [2], highlighting the need for effective food safety communication that reaches everyday consumers.

Poultry is a high-risk food often associated with foodborne illness. The U.S. Department of Agriculture’s Food Safety and Inspection Service has taken regulatory actions to control *Salmonella* in poultry, including improved pathogen reduction standards and proposing classifying *Salmonella* as an adulterant [3]. The Centers for Disease Control and Prevention estimated that about one in every 25 packages of chicken are contaminated with *Salmonella enterica*, a major cause of foodborne illness in the U.S. [4]. Hence, experts recommend cooking chicken to an internal temperature of 165 °F, using separate cutting boards for raw and ready-to-eat foods, and avoiding washing chicken before cooking to prevent cross-contamination [5].

Despite recommendations for safe poultry cooking, social media influencers promote the consumption of raw chicken, claiming it contains more health benefits than fully cooked chicken [6,7,8]. Some individuals expressed that consuming raw chicken helps with dizziness, back pain, and cystic acne, and compared eating raw chicken to eating raw fish, along with other persuasive, unsupported claims [6,7,8]. This situation underscores that anyone can share inaccurate information online and encourage unsafe food practices that increase the risks of foodborne illness.

Consumers are often influenced by food safety information delivered through a variety of communication channels, though they generally seek and trust traditional mass media (e.g., television, radio, newspapers, magazines) more than social media [9,10]. Traditional media offer science-based food safety information from expert sources [11]. Simultaneously, other communication channels, including digital and online media (e.g., news websites, social media, streaming services), have become widely accepted sources of food safety information [12].

Regarding information sources, expert sources are individuals with specialized knowledge in a particular field, gained through academic or scientific research [13]. Experts in government agencies, such as the Center for Disease Control and Prevention, inform consumers about food safety threats, risks, and practices [11]. Social media influencers are individuals who, through popularity online, have potential to influence viewers’ beliefs and attitudes [14]. For example, young adults (aged 18–24) represent the largest current consumer base and spend more time on social media than other generations [15,16], increasing their susceptibility to misinformation. It remains unclear whether consumers are influenced by food safety information disseminated by experts or by social media influencers, suggesting a gap that should be explored.

Although research about food safety communication is growing [17,18,19], the literature on the potential effects of information sources within the context of food safety is limited. Few studies in both national and international contexts have examined how expert versus social media influencer source–message conditions could interact with perceived threat and efficacy in the specific context of food safety practices for raw chicken, and most recent work has focused on pandemic-related rather everyday food safety handling. This study addresses that gap by investigating if information source–message (experts sharing factual information and SMIs sharing misinformation), cognitive, affective, and sociodemographic factors influenced consumers’ intentions to adhere to food safety practices for raw chicken.

## 2. Literature Review

### 2.1. Information Source

An information source could be a person or organization transmitting information about a certain subject or topic [20]. Evidence suggests that source credibility and topic understanding influences consumers’ intentions to protect themselves from food safety risks [21]. Expert sources remain the most trusted when it comes to health-related information [17,21,22,23], and consumers often trust scientists who are specialized in the topic they communicate about [17]. Rembischevski and Caldas [24] emphasized that scientists/universities, medical doctors/health professionals, and nongovernmental organizations were the most reliable sources, with social media influencers being among the least trusted sources. However, this may not always be the case. Settle et al. [25] observed that, when consumers lack trust in the available experts, they turn to trusted social media influencers for health-related information.

Regarding food safety information, Thomas and Feng [22] noted that consumers’ increasing habits of seeking information online make them vulnerable to misinformation that might lead to unsafe practices when handling food. Based on the literature, consumers still rely on expert sources for health-related information. At the same time, emerging literature revealed that social media influencers are becoming popular information sources for health-related information. Hence, the first research question was “Does information source–message affect consumers’ intentions to adhere to food safety practices for raw chicken?” The hypothesis to answer this research question is stated below.

**Hypothesis 1** **(H1).** *Participants who watch a food safety video about raw chicken from an expert source providing factual information will report more intentions to adhere to food safety practices for raw chicken than will participants who watch a social media influencer video providing misinformation.*

### 2.2. Perceived Threat and Perceived Efficacy

The extended parallel processing model (EPPM) explains how fear is used as a communication strategy to promote protective behavior. In this study, protective behaviors were conceptualized as intentions to adhere to food safety practices for raw chicken (i.e., using a thermometer, avoiding eating raw or undercooked chicken, and eating fully cooked chicken). The EPPM was used to assess how perceived threat and perceived efficacy might influence consumers’ intentions to adhere to food safety practices for raw chicken, depending on the information source.

Witte [26] described threat as an external stimulus variable embedded in a message. There are two appraisals described in the EPPM. The first refers to threat, represented by perceived severity and perceived susceptibility (risk). If perceived risk is high, individuals will perceive a high degree of severity (i.e., an individual’s notion of threat seriousness) and susceptibility (i.e., an individual’s notion of risk) to the threat. The second appraisal is perceived efficacy, comprising perceived response efficacy (i.e., an individual’s efficacy notion of actions taken to avoid the threat) and self-efficacy (i.e., an individual’s notion about his or her ability to avoid the threat) [26].

According to EPPM, messages containing fear-based information will lead to protective behaviors if high perceived threat and high perceived efficacy are present [27]. If threat perception is low, the message will be rejected. If individuals perceive a high threat but have a low efficacy perception, they will reject the message or respond with maladaptive behavior out of fear [26].

Liao et al. [28] found consumers with more serious perceptions of foodborne illness threats sought more information and took more protective actions than those with less serious foodborne illness threat perceptions. Thomas and Feng [22] found fear of contracting a disease increased perceived threat, which consequently motivated consumers to take measures to prevent disease. Zhu et al. [29] observed that perceived efficacy significantly contributed to behavior, noting that individuals protected themselves more from threats if they were confident in their abilities to do so. Other researchers [30,31,32] studied this interaction and suggested further investigations to better understand the interplay between variables. Although scholars [29,33,34] studied fear appeals in food safety and crisis communication during the COVID-19 pandemic, limited studies were found for recent and relevant food safety messaging.

In the context of food safety practices for raw chicken, Shumaker et al. [35] observed that consumers who had a serious perceived threat of pathogens in chicken adopted specific food safety practices for raw chicken. Shumaker et al. [35] also reported that participants claimed their cooking behavior was influenced by instructional messages about chicken preparation, highlighting the importance of perceived efficacy appraisal. Rui et al. [36] separately explored the effects of perceived threat, perceived efficacy, and information source (experts vs. social media influencers) on protective behaviors during the COVID-19 pandemic (i.e., staying at home, wearing a face mask, and washing hands). Rui et al. [36] found both perceived threat and perceived efficacy motivated protective behaviors, but expert sources did not encourage protective actions as expected.

Similar to Rui et al. [36], we investigated if perceived threat and perceived efficacy contributed to an indirect pathway between information source–message effect on intentions to adhere to proper food safety practices for raw chicken. The second research question was “Do participants’ intentions to adhere to food safety practices for raw chicken differ when applying perceived threat or perceived efficacy as contributors of an indirect pathway from information source–message?” To answer the second research question, four scenarios were hypothesized.

**Hypothesis 2** **(H2).** *Participants who watch a food safety video about raw chicken from an expert source providing factual information will report stronger perceived threat than those who watch a social media influencer video providing misinformation.*

**Hypothesis 3** **(H3).** *Perceived threat will contribute to an indirect pathway from information source–message and intentions to adhere to food safety practices for raw chicken.*

**Hypothesis 4** **(H4).** *Participants who watch a food safety video about raw chicken from an expert source providing factual information will report stronger perceived efficacy than those who watch a social media influencer video providing misinformation.*

**Hypothesis 5** **(H5).** *Perceived efficacy will contribute to an indirect pathway from information source–message and intentions to adhere to food safety practices for raw chicken.*

### 2.3. Trust in Science

Scholars identified trust in science as an influential factor on consumers’ health-related behavior [37,38,39,40,41]. Man et al. [37] found that, during the COVID-19 pandemic, individuals who trusted science followed expert advice and perceived the virus as more severe. On the contrary, Byrd et al. [38] found consumers who distrusted science were more likely to ignore science-based messages. These same consumers [38] were also more willing to dine out despite expert recommendations and risks during the COVID-19 pandemic. Among consumers who distrusted science, a serious perceived threat was associated with fewer intentions to dine out [38]. These findings align with Entradas [39], who observed that trust in science is strongly associated with perceived threat.

The relationship between trust in science and perceived threat might be more complex, though. From a food safety standpoint, experts claim trust in science signifies trust between stakeholders (e.g., consumers, industries, regulators, and nations) and argue it plays a pivotal role on consumers’ adherence to food safety practices [42]. Consistent with these claims, Kito [41] found consumers’ intentions to go against food safety recommendations by consuming raw chicken were highly associated with their perceived threat and with their trust in those responsible for assessing poultry risks.

Conversely, Wingen et al. [40] found trust in science did not motivate COVID-19 protective behavior (physical distancing, hand hygiene, mask-wearing, and vaccination). In this study, we investigated the interplay between trust in science, perceived risk, and intentions to adhere to food safety practices for raw chicken. The third research question was “Do participants’ intentions to adhere to food safety practices for raw chicken differ when examined by trust in science as a moderator of perceived threat?” These hypotheses answered research question three.

**Hypothesis 6** **(H6).** *When participants with more trust in science watch a factual expert source food safety video about raw chicken, they will report a stronger perceived threat than participants who watch a social media influencer video providing misinformation.*

**Hypothesis 7** **(H7).** *Trust in science will moderate the effect of information source–message on intentions to adhere to food safety practices for raw chicken through perceived threat.*

### 2.4. Food Safety Knowledge, Trust in Information Source, Counterarguing, and Sociodemographic Factors

A lack of knowledge about food safety can make consumers vulnerable to misinformation from misleading sources [43,44]. Soon-Sinclair et al. [45] and Katiyo et al. [46] identified a substantial knowledge gap in consumers’ ability to determine when chicken is safe, often linked to risky food practices. Ballout et al. [47] found a significant association between knowledge and intentions to practice food safety, suggesting that better-informed consumers are more likely to follow recommended food safety practices.

In contrast, Kito [41] found that knowledge was essential for informed decision-making, but it did not influence intentions related to raw chicken food safety practices. Ishra et al. [48] did not observe a direct influence of knowledge on food safety behavior and suggested the possibility of an indirect influence of other factors. These mixed findings indicate a need to continue investigating the role of knowledge on adherence to food safety practices. Food safety knowledge could influence if consumers’ trust in a source influences their intentions, especially on social media where misinformation can be disguised as science-based information [44].

Research has found that trust in information source plays a role in message acceptance and behavioral change [49,50]. According to source credibility theory, the effectiveness of a message varies depending on the extent to which information sources are perceived as experts and trustworthy [51]. For food safety communication, experts are ideal information sources [23,52]. According to Lee et al. [53] and Zhang and Lu [23], individuals often trusted expert sources more than their friends or social media influencers.

On the contrary, Liu and Zheng [54] found that social media influencers perceived as authentic and trustworthy could change consumers’ behaviors. The degree of trust in information sources can influence behavior, especially for those who did not check their source’s accuracy [55]. Scholars studied trust in information sources for food safety information and other health-related topics extensively, often concluding that consumers trusted expert sources [17,21,22,23,40]. However, contrasting studies exist, suggesting there is value in investigating how trust in an information source might influence intentions associated with food safety behavior.

There are cases where consumers resist persuasive messages by counterarguing—mentally creating arguments that rebut information contradicting their beliefs [56]. Walter and Cohen [57] found that persuasive messages with science-based information reduced counterarguing because it was difficult for consumers to contradict expert statements. Building on the elaboration likelihood model, Feng et al. [58] found that the message deliverer might influence message resistance and processing. Consequently, the extent to which consumers counterargue with health information may be associated with how information is presented [59]. For example, presenting messages as stories, whether science-based or not, was associated with less counterarguing [59]. Given that social media influencer sources often use storytelling strategies to share information [60], these findings highlight an opportunity to study counterarguing further.

Previous research has demonstrated that sociodemographic characteristics (e.g., age, gender, education, income, and cultural background/race) may play a role in consumers’ adherence to food safety practices [61,62,63,64,65,66]. For example, in the U.S., racial minority groups bear the highest rate of diet-related chronic diseases and often lack access to food safety education, which might influence their adherence to food safety practices [66]. In a study conducted in Houston, Texas, low-income consumers from Hispanic, Latino, or Mexican American households were less likely to use a food thermometer at home [62].

Similarly, in the retail industry, ethnic-operated restaurants (Hispanic, Asian, or Italian) received more violations related to improper holding temperatures or inadequate cooking than non-ethnic-operated restaurants [61]. Concerning raw chicken, unsafe practices, including using a thermometer, varied by race, income, and education [62,67,68]. Scholars also suggested that Hispanic and Black individuals were more likely than White individuals to base health decisions on social media influencer content [69], and Hispanic individuals tended to interact with visual misinformation more than other races [70].

Gender and education level played a role on intentions to follow recommended food safety practice [64,68]. Individuals with higher than high school education were expected to adhere to food safety practices more often than those with less education [64]. According to Miftari et al. [64], highly educated females were more concerned about food safety risks and adherence to food safety practices than males. Gender was also related to preferred sources of information, as females were more likely than males to obtain food safety information from social media [64].

Regarding age, evidence is more mixed. Sezer [71] found individuals aged 18–24 were more likely to handle and prepare safe food than those aged 25–30. Yi and Choi [65] reported contrasting results that indicated adults in their 40–60s reported higher intentions to practice food safety than did younger adults in their 20s [65]. Older adults took more precautions to protect themselves from foodborne illness than did younger adults [65]. Older adults’ preferred traditional media, government, or health professionals as information sources, while younger adults often relied on the internet and social media for food safety information [65]. Taken together, these findings suggest value in continuing to explore the role of sociodemographic factors on intentions to adhere to food safety practices.

Based on our literature review, the fourth research question was “Do participants’ intentions to adhere to food safety practices for raw chicken differ when examined with food safety knowledge, trust in information source, counterarguing, and selected sociodemographic factors as moderators?” The following hypotheses answered the fourth research question.

**Hypothesis 8** **(H8).** *Food safety knowledge will moderate the effect of information source–message on intentions to adhere to food safety practices for raw chicken.*

**Hypothesis 9** **(H9).** *Trust in information source will moderate the effect of information source–message on intentions to adhere to food safety practices for raw chicken.*

**Hypothesis 10** **(H10).** *Participants who watch a food safety video about raw chicken from a social media influencer source providing misinformation will report more counterarguing than participants who watch an expert video providing factual information.*

**Hypothesis 11** **(H11).** *Counterarguing will moderate the effect of information source–message on intentions to adhere to food safety practices for raw chicken.*

**Hypothesis 12** **(H12).** *Sociodemographic factors (i.e., age, gender, education, income, and race) will moderate the effect of information source–message on intentions to adhere to food safety practices for raw chicken.*

## 3. Materials and Methods

### 3.1. Study Design

This study is part of a larger project where similar methods were described in the raw milk and collard greens contexts [72]. A posttest-only, between-subject, quasi-experimental cross-sectional survey [73] was used to study potential influencing effects of information source on consumers’ intentions to adhere to food safety practices for raw chicken. An advantage of between-subject designs is that participants only view one condition, which helps reduce the threat of a carry-over effect [74]. This study design was quasi-experimental because assignment to experimental conditions was preference-based (self-selection based on topic of interest), preventing random assignment as an experimental condition [75].

### 3.2. Experimental Conditions

Food safety messages were presented to consumers through short videos (1–2 min) because short videos are more effective in retaining consumers’ attention to science-based messages [76,77,78]. Participants who self-selected into the raw poultry topic were randomly assigned, using a computer-generated randomization procedure with equal allocation, to one of two conditions: (1) expert source video, or (2) social media influencer video. Participants who selected “no preference” were assigned to view the control video only. After video assignments, *n* = 215 participants were assigned to a food safety video from an expert, *n* = 205 watched a social media influencer video, and *n* = 307 were assigned to an exploratory handwashing control video. After watching the food safety video, participants answered questions about food safety knowledge, trust in science, trust in information source, counterarguing, perceived threat, efficacy, and sociodemographic information.

Food safety videos were created with HeyGen [79], a generative artificial intelligence (GAI) tool, which was based on researcher-developed scripts. According to Leiker et al. [80], Netland et al. [81], and Shu et al. [82], videos generated by AI have the same learning outcomes as videos recorded traditionally. Using HeyGen (2025) allowed the creation of more cost-effective and static experimental stimuli to maintain consistency in video message delivery and style.

Two source personas were developed, an expert and a social media influencer, for treatment videos. Costello and Rutherford’s [83] definition of an expert represented expert sources. Experts were individuals with demonstrated food safety knowledge and experience, including food safety extension agents, food scientists with a food safety specialization, and food safety public health officials. Social media influencers were defined as individuals with a social media presence who may influence others’ actions through their content [84]. Social media influencers had no credentials in food safety and used personal beliefs and experiences to communicate their messages. Each persona’s role was reflected in a brief introduction before information was presented.

To maintain consistency, reduce potential gender and racial biases, and to reflect the U.S. population’s diversity [85], generated personas were male and female of Hispanic, White, and Black races (the three most prevalent racial groups in the U.S.) [86,87,88,89,90]. Each persona was an avatar selected from the HeyGen stock library.

We adjusted voices for each avatar to ensure personas sounded natural and realistic. For the influencer personas to sound authentic, we extracted audio from publicly available videos using Restream [91] and cloned voices in HeyGen. We wrote video scripts based on the literature about consuming raw chicken [4,6,7,8]. Video scripts were developed without the use of AI. For expert and exploratory control videos, content was aligned with food safety standards recognized by the Centers for Disease Control and Prevention and Food and Drug Administration, which are U.S. institutions that oversee food safety. For the social media influencer source, video content was derived from misinformation based on personal beliefs and experiences and did not align with food safety standards [92].

### 3.3. Instrumentation

We developed a cross-sectional survey instrument to present consenting participants with food safety videos about raw chicken. The Marketing Systems Group distributed the online instrument to consumers in the U.S. First, the instrument required participants’ consent before entering the study. If they agreed to the study conditions, participants were directed to the next section. If they did not agree, they left the instrument. The first section contained a qualifying question about age, using categories from the U.S. Census Bureau [86] (i.e., 18–24, 25–34, 35–44, 45–54, 55–64, and 65+). Those who were not above 18 could not participate and were excused from the study. An attention check question was used to identify inattentive respondents. We asked participants about their frequency of poultry consumption (six-point scale for most days (6) to never (1)), internet use for health-related information, and social media influencer content (seven-point scale for most days (7) to never (1)).

We measured food safety knowledge with 12 multiple-choice items adapted from Byrd-Bredbenner et al.’s [93] validated food safety knowledge scale. After conducting a confirmatory factor analysis that determined poor factor loadings, we removed six items and retained the remaining six for our analysis. The number of items used to measure knowledge are presented in Table 1.

We asked participants about their trust in science with seven items adapted from Rumble et al. [94], based on the National Science Board’s Science and Engineering Indicators Report [95]. The seven items to measure trust in science were (1) development in science makes society better; (2) scientists contribute to the well-being of society; (3) scientific research should be supported by the federal government; (4) scientific research is essential for improving the quality of human lives; (5) new technology used in medicine allows people to live longer; (6) new technology used in medicine allows people to live better lives; and (7) overall, modern science does more harm than good (seven-point scale of strongly agree (7) to strongly disagree (1)). After conducting a confirmatory factor analysis to evaluate the scale, item 7 was removed from data analysis.

We asked a ranking question to assign participants to a food safety video: “If you search the internet for food safety videos for these food groups, which group would rank first and which one last in your searches?” Participants ordered the four options (poultry, leafy greens, dairy, and no preference) from most to least preferred. Participants who ranked poultry first were assigned to food safety videos about raw chicken. Participants who selected “no preference” were assigned to the control video.

Before watching the video, participants were prompted: “You will now watch short videos about food safety. Some content might be AI-generated.” After watching food safety videos, participants answered questions about perceived threat, perceived efficacy, trust in information source, counterarguing, intentions to adhere to food safety practices, an attention check, and sociodemographic information.

We evaluated perceived threat and perceived efficacy with items originally developed by Witte [96] and adapted by Shi and Smith [97]. The items used to measure perceived threat and perceived efficacy are listed in Table 2 and Table 3 (seven-point scale of strongly agree (7) to strongly disagree (1)).

Participants were asked to evaluate their trust in information source with five items: (1) trustworthiness; (2) reliability; (3) dependability; (4) honesty; and (5) sincerity (seven-point differential scale based on their perceptions of the speaker in the video (e.g., untrustworthy/negative (1) to trustworthy/positive (7)). These items were adapted from Chambers [17], originally developed by Ohanian [98].

We assessed counterarguing using Yan’s [99] validated scale, originally adapted from Silvia [100]: (1) while watching the video, I was criticizing what the speaker was saying; (2) while watching the video, I was thinking of points that went against the speaker’s argument; and (3) while watching the video, I was feeling skeptical of the speaker’s arguments (seven-point scale for strongly agree (7) to strongly disagree (1)).

We measured intentions to adhere to food safety practices for raw chicken with three items: (1) based on the video you watched, how often would you avoid eating raw or undercooked chicken in the future?; (2) based on the video you watched, how often would you use a thermometer to check if your chicken has reached the recommended safe temperature?; and (3) based on the video you watched, how often would you eat fully cooked chicken in the future? (five-point scale for always (5) to never (1)). For the exploratory control condition on handwashing, we measured intentions with the following items: (1) based on the video you watched, indicate how often would you wash your hands before preparing food in the future?; (2) based on the video you watched, how often would you wash your hands before eating in the future?; and (3) based on the video you watched, how often would you wash your hands after touching an animal? (five-point scale for always (5) to never (1)). These items were adapted from Madjdian et al. [101], originally developed by Witte [96].

To ensure participants were answering intentionally, we asked participants to select their answer from the following options: YouTube, Instagram Reels, X (formerly Twitter), Facebook, or Other (please specify). The survey continued only for participants who passed the attention check.

The final section included multiple-choice demographic questions [102]. Participants reported their gender identity (male, female, other, prefer not to respond); educational level completed (some high school, high school graduate, some college, associate’s degree, bachelor’s degree, master’s degree, doctoral degree, professional degree, and trade/technical/vocational training); average annual household income (less than $10,000, $10,000 to $24,999, $25,000 to $34,999, $35,000 to $74,999, $75,000 to $99,999, $100,000 to $149,999, $150,000 to $249,999, $250,000 or more, prefer not to respond); and cultural background/ethnicity (White or Caucasian, Black or African American, Hispanic or Latino, Asian, some other race, ethnicity, or origin, or prefer not to respond). We asked participants if they were the primary grocery shoppers in their household and if they had children or dependents under the age of 18 living at home.

### 3.4. Validity and Reliability

For face and content validity, a panel of subject matter experts in food safety and social science evaluated the instrument [103]. We conducted a pilot study at [Blinded] University with a sample of undergraduate and graduate students to establish instrument reliability [104]. We conducted a confirmatory factor analysis (CFA) and calculated a McDonald’s omega to measure construct reliability (CR) [105] on the final data set.

We assessed factor loadings, convergent and discriminant validity, and overall model fit [106]. The food safety knowledge scale’s reliability was assessed using the Kuder–Richardson Formula 20 (KR-20) [107]. All scale reliability coefficients were above the acceptable threshold (>0.70), except for food safety knowledge, which was below the acceptable thresholds of 0.50 for KR-20 and 0.70 for CR. These low values could be attributed to content heterogeneity, given that questions measured different food safety domains [108,109,110]. The low food safety knowledge scale’s reliability is noted as a limitation.

### 3.5. Data Collection and Sample

The target population was U.S. consumers over 18 years old. The accessible population consisted of consumers who participated in surveys for Marketing Systems Group. The population of interest was adults between the ages of 18 to 24 residing in the U.S. in 2025. Gen Z individuals often trust social media influencers for food choices and cooking advice, which may increase their susceptibility to misinformation. Understanding young adults’ food safety practices is important, given that most young adults tend to use social media for food safety information [111].

We conducted a Monte Carlo simulation with Mplus version 9 software to estimate sample size. The results indicated that a statistical power of 0.8, the typical adequate power in behavioral sciences [112,113], could be detected with a total sample of *N* = 200. A sample size of *N* = 200 also aligned with the recommendation of sample size for SEM [114,115,116].

We collected data from 15 to 18 December 2025, yielding more than 700 respondents (Table 4). The panel of participants, a non-probability, opt-in sample, may have received incentives from the survey distribution partner, although the exact incentive amount or type is unknown. Participants’ sociodemographic characteristics are described in Table 4. The largest group (44.4%) was adults aged 65 and over. Among participants who reported gender (*n* = 653), ~60% identified as female. Overall, 31.4% of participants reported never using the internet to search for food safety information, and 29.8% never interacted with content from social media influencers. More than one-half (51%) indicated consuming poultry several times a week. Among participants who reported education level (*n* = 653) and income (*n* = 652), the largest group had at least a bachelor’s degree (26.8%), with a reported (34%) income of $35,000 to $74,999 a year. Most (78.5%, *n* = 652) identified as White, were the primary grocery shoppers in their households (89%, *n* = 653), and did not have children under the age of 18 living at home (79.5%, *n* = 652). Among those who had children under the age of 18 living at home, 65.4% (*n* = 133) indicated having one child at home.

### 3.6. Data Analysis

We analyzed data using SPSS version 31 for descriptive statistics, assumption checks, and multiple regression analyses. We used Mplus version 9 for structural equation modeling. The dependent variable of interest was intentions to adhere to food safety practices for handling and preparing raw chicken. The independent variable was information source–message. Perceived threat and perceived efficacy were modeled as contributors to an indirect pathway from information source–message and intentions to adhere to food safety practices for raw chicken. Food safety knowledge, trust in science, trust in information source, counterarguing, and sociodemographic characteristics were modeled as moderators. Missing data in the regression analyses were handled through listwise deletion; cases with missing data on any variable included in a model were excluded from that model. For the analyses in Mplus, missing values were coded as −99. A Shapiro–Wilk test indicated that normality of residuals was violated (*p* < 0.01). High kurtosis (>7) and skewness values (>2) also indicated a non-normal distribution [117]. A scatterplot of standardized residuals versus standardized predicted plots indicated that homoscedasticity assumptions were not met [118].

Descriptive statistics were used to describe variable means, standard deviations, and correlations. We conducted one-sample Wilcoxon signed rank tests to compare each variable mean to its respective scale midpoint. We cross-tabulated the ratings from information source trust scale to better describe the distribution of trust ratings across participants. We tested hypotheses 1–11 (H1–H11), except H10, using structural equation modeling (SEM). We conducted a confirmatory factor analysis for the measurement model to assess model fit and evaluate indicators of convergent and discriminant validity using the WLSMV estimator because the food safety knowledge indicators were categorical. Structural models A and B were estimated using MLR due to the non-normality of data. For H10, we conducted an independent-sample *t*-test using SPSS version 31. Among the model fit indices, the root mean square of error approximation indicated how far a hypothesized model is from a perfect model. The standardized root mean square residuals assessed how well the hypothesized model represents the associations between variables. The comparative fit index (CFI) and Tucker–Lewis index compare the hypothesized model with a baseline model [119,120].

To test hypothesis 12 (H12), we conducted a multiple linear regression including interaction terms to examine moderation effects. Age was dummy-coded (1 = 35 and older, referent; 0 = all others); education (1 = associate’s, bachelor’s, trade/vocational training, referent; 0 = all others); income (1 = $35,000–$74,999, referent; 0 = all others); and race (1 = White or Caucasian, referent; 0 = all others). In Model 1, we tested the main effects of each sociodemographic variable. In Model 2, we included interaction effects between the source and sociodemographic variables to test for moderation effects, and we evaluated the change in explained variance between models.

### 3.7. Limitations

Some limitations include threats to internal validity such as selection bias, history, and maturation [121]. To control selection bias, we collected baseline traits before assignment. Participants were unaware of their assignment, and there was randomization within preference [122,123,124]. Another limitation was our handwashing control condition comparisons, which could not be interpreted as isolated source–message effects because the control differed in selection pathway, video topic, message content, and outcome context. We also note that because heteroscedasticity was present in our data, results from our regression models must be interpreted with caution.

Reliance on self-reported data is a limitation of cross-sectional surveys because it may not represent participants’ true answers, as well as participants’ responses being reflective of those in the MSG participant pool [121].

Another uncontrolled limitation was the use of AI videos, because there could be potential bias if participants perceived AI-generated videos differed from traditional videos with real people. Because most participants were 65 and older with only ~13% aged 18 to 34, the findings should not be generalized to young adults. This study addressed intentions to adhere to food safety practices for handling and preparing raw chicken, which reduces generalizability to other contexts.

## 4. Results

### 4.1. Descriptive Statistics for Study Variables

To describe our data before testing the hypotheses, we conducted descriptive analyses. To compare participants’ scores to the scale’s midpoint (neutral), we conducted one-sample Wilcoxon signed-rank tests. Participants’ intentions to adhere to food safety practices for handling and preparing raw chicken were significantly above neutral across all food safety video conditions, *Z* = 15.87, *p* < 0.001, *r* = 0.86 to *r* = 0.89. Participants reported elevated trust in science (*M* = 5.97, *SD* = 1.03), relative to the midpoint, *Z* = 23.10, *p* < 0.001, *r* = 0.86. Food safety knowledge scores were above the scale’s midpoint (*M* = 3.80, *SD* = 1.44), *Z* = 12.80, *p* < 0.001, *r* = 0.47.

After watching their assigned food safety video, participants perceived threat was significantly above the neutral midpoint; the greatest observed mean was experts’ food safety videos (*M* = 6.64, *SD* = 0.71), *Z* = 13.09, *p* < 0.001, *r* = 0.89. Participants perceived efficacy was significantly greater than the neutral midpoint, with the greatest identical means observed for food safety videos from experts (*M* = 6.63, *SD* = 0.57), *Z* = 12.96, *p* < 0.001, *r* = 0.88 and control (*M* = 6.63, *SD* = 0.71), *Z* = 15.67, *p* < 0.001, *r* = 0.89. Participants indicated elevated trust in all information sources when compared to the neutral midpoint, with the greatest mean observed for trust in expert sources (*M* = 6.25, *SD* = 1.06), *Z* = 12.74, *p* = 0.000, *r* = 0.87. Participants counterargued statements from social media influencers’ food safety videos the most (*M* = 4.03, *SD* = 2.15), significantly above the neutral perspectives, *Z* = 3.21, *p* < 0.005, *r* = 0.23 (Table 5).

Participants assigned to expert food safety videos were more likely to perceive the speaker as dependable, honest, reliable, sincere, and trustworthy (7 = positive endpoint), and less likely to perceive the speaker as undependable, dishonest, unreliable, insincere, and untrustworthy (1 = negative endpoint). Participants who watched food safety videos from influencers showed the opposite pattern. We cross-tabulated the ratings from the scale measuring trust in information source. We assessed the negative (1) and positive (7) endpoints to describe any differences in column proportions across video conditions using pairwise *z*-tests with Bonferroni-adjusted *p* values at a 0.05 significance level.

Among participants who watched food safety videos from experts (*n* = 215), they indicated the speaker was dependable (57.67%), honest (60.47%), reliable (58.14%), sincere (56.74%), and trustworthy (60%). Those who watched food safety videos from influencers (*n* = 205) perceived the speaker as dependable (33.17%), honest (35.61%), reliable (34.63%), sincere (39.02%), and trustworthy (34.63%). The proportion of extreme positive and extreme negative trust ratings did not differ significantly between expert and control conditions (*p* > 0.05). In contrast, the proportion of extreme positive and negative trust ratings in the social media influencer condition differed significantly from expert and control conditions (*p* < 0.05) (Table 6).

In exploratory comparisons involving the handwashing control condition, participants in the control group were more likely than participants in the social media influencer condition to rate the speaker as dependable, honest, reliable, sincere, and trustworthy, and less likely to rate the speaker as undependable, dishonest, unreliable, insincere, and untrustworthy. Ratings in the handwashing control and expert conditions did not significantly differ.

Positive significant correlations existed between intentions to adhere to raw chicken food safety practices and food safety knowledge (*r* = 0.204, *p* < 0.001), trust in science (*r* = 0.134, *p* = 0.001), perceived risk (*r* = 0.459, *p* < 0.001), perceived efficacy (*r* = 0.496, *p* < 0.001), and trust in information source (*r* = 0.282, *p* < 0.001). A negative significant correlation existed between intentions to adhere to raw chicken food safety practices and counterarguing (*r* = −0.307, *p* < 0.001) (Table 7).

Positive correlations existed between intentions to adhere to raw chicken food safety practices and age (*r* = 0.459, *p* < 0.001). Negative significant correlations existed between intentions to adhere to raw chicken food safety practices and income (*r* = −0.109, *p* < 0.001). No statistically significant relationships existed between intentions and gender, education, or race (Table 8).

To test hypothesis 10 (H10), we conducted an independent-sample *t*-test comparing counterarguing between the SMI–misinformation and expert–factual conditions. Participants in the SMI–misinformation condition reported significantly more counterarguing (*M* = 4.03, *SD* = 2.15, *n* = 205) than participants in the expert–factual condition (*M* = 2.15, *SD* = 1.39, *n* = 215), mean difference = 1.88, 95% CI [1.53, 2.23], *t*(346.83) = 10.59, *p* < 0.001, Cohen’s *d* = 1.04, 95% CI [0.84, 1.25].

### 4.2. Measurement Model

Before conducting SEM to answer all our research questions, we first conducted a CFA to assess the measurement model fit as well as validity and reliability indicators. To assess convergent validity, we evaluated average variance extracted (AVE) and CR. Convergent validity can be affected by factor loadings below 0.50, which indicates items may not have measured the construct appropriately [125,126]. However, if the sample is at least *N* = 350, items with factor loadings of 0.3 can be retained [125,127]. We excluded seven items with factors below 0.3 (one item from trust in science and six from food safety knowledge) [128]. The food safety knowledge scale’s AVE was below the threshold of 0.5, but because the CR was over 0.6, the construct’s convergent validity was adequate [129]. The items measuring perceived threat, perceived efficacy, trust in science, trust in information source, and counterarguing significantly loaded on their constructs (*p* < 0.001), and met AVE and CR thresholds of >0.50 and >0.70, respectively [119,120,130,131]. Intentions to adhere to food safety practices significantly loaded on their construct (*p* < 0.001) and met CR thresholds but not AVE. This low AVE is noted as a limitation (Table 9).

The measurement model was respecified before testing the structural model. The root mean square error of approximation = 0.031, standardized root mean square residual = 0.036, CFI = 0.950, and TLI = 0.945. Hence, the model fit for the respecified model was acceptable [119,120,130,131] (Table 10).

### 4.3. Structural Models

#### 4.3.1. Structural Model A

Hypotheses 1–5 (H1–H5) were tested with structural model A. We evaluated if information source–message had a direct and indirect effect on intentions to adhere to raw chicken food safety practices in structural model A. The model fit indices indicated a good model fit for structural model A: RMSEA = 0.045, SRMR = 0.031, CFI = 0.955, and TLI = 0.945. Although the chi-square statistic was significant (χ2 (111) = 273.849, *p* < 0.001), the literature states that the chi-square test often rejects the null hypothesis, especially with large sample sizes. We reported additional information from the model fit indices, which suggested the hypothesized structural model A was acceptable and feasible [129] (Table 11).

Structural model A accounted for 56.6% of the variance in participants’ intentions to adhere to food safety practices when handling and preparing raw chicken (Figure S1), *R*^2^ = 0.566. Exploratory comparisons with the handwashing control reveal that the expert–factual (β = −0.211, *p* < 0.001) and social media influencer–misinformation conditions (β = −0.300, *p* < 0.001) had significant, negative direct effects on intentions. However, it is important to note that these control contrasts should not be interpreted as isolated source–message effects. The total effects from the social media influencer–misinformation condition were β = −0.374, 95% CI [−0.497, −0.251] and from the expert–factual information condition were β = −0.160, 95% CI [−0.262, −0.058]. Participants who watched a food safety video from an expert source reported stronger perceived threat (β = 0.344, *p =* 0.001) and stronger perceived efficacy (β = 0.265, *p* < 0.001) than those in the influencer and control conditions. Perceived threat (β = 0.047, *p =* 0.033) and perceived efficacy (β = 0.068, *p =* 0.007) contributed to an indirect pathway from information source–message on intentions to adhere to food safety practices for handling and preparing raw chicken (Table 12).

#### 4.3.2. Structural Model B

Hypotheses 6–11 (H6–H11) were tested in structural model B. We evaluated if food safety knowledge, trust in science, trust in information source, and counterarguing moderated the effects of information source on intentions to adhere to raw chicken food safety practices (Table 13). Model fit indices indicated a good fit for structural model B, RMSEA = 0.038, SRMR = 0.069, CFI = 0.955, and TLI = 0.946. The chi-square statistic was significant, (χ2 (135) = 274.697, *p* < 0.001).

Structural model B accounted for 47.6% of the variance in participants’ intentions to adhere to food safety practices for raw chicken (Figures S1 and S2), *R*^2^ = 0.476. Trust in science had a positive, significant direct effect on perceived threat (β = 0.364, *p* < 0.001) across all video conditions, but it did not moderate the effect of information source–message on intentions through perceived threat. Food safety knowledge and trust in information source had significant, positive direct effects on intentions, but no significant moderation effects of information source–message were found. Likewise, we found no significant direct or moderation effects of counterarguing on intentions to adhere to food safety practices for raw chicken (Table 14).

### 4.4. Multiple Regression Analyses

The main effects of sociodemographic variables on participants’ intentions to adhere to food safety practices for handling and preparing raw chicken are described in Table 15. Information source–message conditions were our central independent variables. Model 1 accounted for approximately 8% of the variance in intentions, *R*^2^ = 0.08, Adj. *R*^2^ = 0.06, *F* (12, 571) = 4.134, *p* < 0.001. Relative to the exploratory control source, which does not represent isolated source–message effects, the social media influencer source (β = −0.277, *p* = 0.000), and expert source (β = −0.095, *p* = 0.037) were associated with fewer intentions to adhere to food safety practices for handling and preparing raw chicken. Participants who identified as Hispanic or Latino reported fewer intentions to adhere to food safety practices (β = −0.087, *p* = 0.041) compared to those who identified as White. The remaining socio-demographic variables had no significant effects.

In Model 2, interaction effects of sociodemographic variables on intentions to adhere to food safety practices for handling and preparing raw chicken were added to the main effects. The results are presented in Table 16. Compared to Model 1, this model increased the variance explained in intentions by approximately 4%. Model 2 accounted for about 12%, *R*^2^ = 0.116, Adj. *R*^2^ = 0.065, *F* (32, 551) = 2.259, *p* < 0.001. Adding interaction effects changed the main effect from social media influencer sources (β = −0.292, *p* = 0.008) on intentions, compared to the exploratory control source, and the expert source did not significantly affect intentions. In Model 2, males reported more intentions to adhere to food safety practices for handling and preparing raw chicken (β = 0.168, *p* = 0.011) when compared to females. There were no significant interaction effects, indicating that no sociodemographic factors moderated the effect of information source–message on participants’ intentions to adhere to food safety practices for handling and preparing raw chicken.

Table 17 provides conclusions for each hypothesis, either rejection or failure to reject the null hypothesis.

## 5. Discussion

We investigated if information source (i.e., experts providing factual information, social media influencers providing misinformation) and cognitive and affective factors influenced consumers’ intentions to adhere to food safety practices for raw chicken. Participants reported strong intentions to adhere to food safety practices for raw chicken. Participants who watched food safety videos about raw chicken from an expert source reported stronger perceived threat and perceived efficacy than those who watched an influencer video. These findings are consistent with Rui et al. [36] who found that expert sources were often associated with more perceived threat and perceived efficacy.

Perceived threat and perceived efficacy directly and indirectly influenced intentions to adhere to food safety practices for raw chicken. These findings support Thomas and Feng [22] who observed that when consumers perceived strong food safety risks and efficacy, they were more likely to take action to protect themselves from these risks. In the context of raw chicken food safety, our findings aligned with Shumaker et al. [35] who found that instructional videos encouraged adherence to food safety practices due to perceived efficacy. The modeled path between perceived threat, perceived efficacy, and information source–message explained more than half of the variance in consumers’ intentions to adhere to food safety practices for raw chicken. These findings suggest that perceived threat and perceived efficacy play essential roles in thought processing of food safety messages.

Participants with more trust in science reported more perceived threat, confirming the findings of Byrd et al. [38] and Entradas [39] who found trust in science is generally associated with a stronger risk perception. However, trust in science did not indirectly moderate the effect of information on intentions via perceived threat. In support of Wingen et al. [40], the association between trust in science and perceived threat may not always be related to stronger intentions. Most recent research [24,37,38,39,40] on trust in science and food safety has focused on pandemic-related contexts, which might possibly explain why trust in science might be less connected to everyday food safety decisions.

Food safety knowledge directly affected intentions to adhere to food safety practices for raw chicken, but it did not moderate the effect of information source–message on intentions. These findings support Ballout et al.’s [47] findings that highlighted knowledgeable consumers were more likely to practice food safety while contradicting those of Kito [41] who found no association between food safety knowledge and intentions to consume raw chicken. We speculate that because Kito’s [41] study was set in Japan, cultural components might have influenced those findings. A possible explanation for the lack of a moderating effect of knowledge could be attributed to intentions; knowledge may be an insufficient contributor to moderate the effect of information source–message. These findings support Ishra et al.’s [48] stance that knowledge can be a significant mediator of factors (i.e., attitude), but knowledge alone might not be sufficient to moderate the effects of other factors.

Trust in information source directly affected intentions to adhere to food safety practices for raw chicken, which is congruent with prior work [17,21,22,23]. However, trust in information source did not moderate the effect of information source–message on intentions. Like knowledge, a potential explanation for the insignificant moderation effect of trust in information source could be that trust in source alone is not strong enough to moderate the effect of information source–message on intentions [48].

The multiple regression model indicated that participants who identified as Hispanic or Latino reported less intention to adhere to food safety practices for raw chicken than those who identified as White. These findings align with Carstens [62] and Boutros and Roberts [61] who found that Hispanic or Latino individuals were less likely to practice food safety than White individuals. However, when we added interaction terms to the regression model, no sociodemographic variables moderated the effect of information source–message on intentions. One possible explanation is attributed to a homogenic sample unable to detect these effects. Therefore, sample characteristics are a study limitation. These findings underscore the complexity of isolating the effects of sociodemographic factors because they may be interrelated. Hence, it is challenging to conclude that one single factor made a unique contribution to explain participants’ intentions to adhere to raw chicken food safety practices. Scholars should address the role of sociodemographic factors with larger and more diverse participant groups.

Counterarguing was significantly greater for the social media influencer–misinformation condition than the expert–factual condition, supporting Cohen [57], who found that persuasive messages with science-based information reduced counterarguing because it was difficult for consumers to contradict expert statements. We found no significant direct or indirect effects of counterarguing on intentions to adhere to food safety practices for raw chicken. A possible explanation, beyond the scope of this study, could be that message framing helped shape participants’ resistance to the message, as observed by Feng et al. [58] and Dudley et al. [59].

The findings from this study contribute to the growing food safety communication literature by showing that perceived threat and perceived efficacy help explain how information source–message could influence intentions to adhere to food safety practices for raw chicken. In the future, scholars should investigate actual behavior rather than intentions by conducting observational studies with observable food safety behaviors. Observational studies could track thermometer use, cross-contamination, handwashing, and other recommended food safety practices.

The use of AI-generated videos was an efficient way to test information source–message effects, but more research is needed to test if audiences respond differently to AI-generated vs. human speakers. With AI, the speaker’s characteristics can be modified easily. This ability offers an opportunity to study factors such as the speaker’s individual characteristics or homophilic characteristics influencing message acceptance and intentions to follow the speaker’s advice.

We recommend food safety messages be designed in accordance with Witte’s [26] extended parallel processing model, which is to provide clear explanations of food safety risks and achievable steps to avoid foodborne illnesses. We recommend conducting an audience segmentation to identify which populations are more at risk of contracting foodborne illness, particularly for those who handle and consume chicken frequently. Audience segmentation could help identify susceptible consumers, guide the design of food safety communication about raw chicken, and allow replication within other high-risk food contexts. A strategy to combat misinformation on social media could be to foster collaboration between credible social media influencers and experts/public health organizations to design and deliver evidence-based food safety education messages. Social media sources should include disclosures about health claims, especially claims that contradict expert food safety recommendations.

This study could be replicated while including questions about participants’ prior experiences with foodborne illnesses, which might affect how they respond to messages that increase foodborne illness risks. Other message conditions could also be tested (i.e., social media influencers sharing factual information vs. experts spreading misinformation) to evaluate differences between source and message effects. In addition, as AI fatigue continues, studying differences in message effectiveness between conventional and AI-generated videos is valuable.

## 6. Conclusions

Hypotheses 2, 3, 4, 5, and 10 were supported. Participants who watched a food safety video about raw chicken from an expert source who provided factual information reported stronger perceived threat and perceived efficacy than those who watched a social media influencer who provided misinformation. Perceived threat and perceived efficacy contributed to an indirect pathway from information source–message and intentions to adhere to food safety practices for raw chicken. Counterarguing was greater for social media influencer–misinformation than for expert–factual information.

Our findings should be interpreted with caution. We note that we measured self-reported intentions to adhere to food safety practices for raw chicken rather than observed behavior. Effects from the source and message were combined and individual effects were not isolated. In addition, the handwashing control group was exploratory because it was self-selected and used different contexts than raw chicken. Because we measured our main constructs at the same post-exposure time point, our sample was not representative of young adults, and included a non-probability, opt-in panel, caution must be warranted against generalizing our findings.

## Figures and Tables

**Table 1:** Selected food safety knowledge items [93].

Food Safety Domain	Items Adapted	Items Retained
Cross-contamination prevention/disinfection procedures	3	1
Safe times/temperatures for cooking/storing food	3	3
Foods that increase the risk of foodborne illness	2	0
Common food sources of foodborne disease pathogens	3	1
Groups at greatest risk for foodborne disease	1	1

**Table 2:** Items used to measure perceived threat.

Context	Items	Threat Component
Raw Chicken	I am at risk of getting sick if I eat raw chicken (1)	Susceptibility
Raw Chicken	I am susceptible to getting sick if I eat raw chicken (2)	Susceptibility
Raw Chicken	I can get sick from eating raw chicken (3)	Susceptibility
Raw Chicken	Eating raw chicken poses a serious threat of foodborne illness (4)	Severity
Raw Chicken	Eating raw chicken can be harmful to my health (5)	Severity
Raw Chicken	Foodborne illness caused by eating raw chicken can have severe health consequences (6)	Severity
Handwashing	I am at risk of getting sick if I don’t wash my hands (1)	Susceptibility
Handwashing	I am susceptible to getting sick if I don’t wash my hands (2)	Susceptibility
Handwashing	I can get sick from not washing my hands (3)	Susceptibility
Handwashing	Not washing my hands poses a serious threat of foodborne illness (4)	Severity
Handwashing	Not washing my hands can be harmful to my health (5)	Severity
Handwashing	Foodborne illness caused by not washing my hands can have severe health consequences (6)	Severity

**Table 3:** Items used to measure perceived efficacy.

	Items	Efficacy
Raw Chicken	I can follow the correct steps to cook chicken thoroughly (1)	Self-
Raw Chicken	It is easy for me to follow safe cooking practices when preparing chicken (2)	Self-
Raw Chicken	I can properly cook chicken to protect myself from getting sick (3)	Self-
Raw Chicken	Cooking chicken thoroughly protects me from getting sick (4)	Response
Raw Chicken	Cooking chicken thoroughly helps protect me from getting sick (5)	Response
Raw Chicken	Cooking chicken thoroughly is an effective practice that protects me from getting sick (6)	Response
Handwashing	I can wash my hands (1)	Self-
Handwashing	It is easy for me to wash my hands (2)	Self-
Handwashing	I can wash my hands to protect myself from getting sick (3)	Self-
Handwashing	Choosing to wash my hands protects me from getting sick (4)	Response
Handwashing	Choosing to wash my hands helps protect me from getting sick (5)	Response
Handwashing	Washing my hands is an effective practice that protects me from getting sick (6)	Response

**Table 4:** Sociodemographic characteristics of participants (*N* = 727).

Characteristics	*n*	*f*	%
Age ^a^	727		
65 and over		323	44.40
55–64		141	19.40
35–44		93	12.80
45–54		88	12.10
18–34		82	12.80
Gender	653		
Female		390	59.70
Male		262	40.10
Prefer not to respond		1	0.10
Food Safety Information Online Searches	727		
Never		228	31.40
Once every few months		184	25.30
Once a month		81	11.10
Several times a week		66	9.10
Most days		63	8.70
Once every two weeks		55	7.60
Once a week		50	6.90
Interaction with Content from Social Media Influencers	727		
Never		217	29.80
Most days		166	22.80
Several times a week		147	20.20
Once every few months		75	10.30
Once a week		64	8.80
Once a month		30	4.10
Once every two weeks		28	3.90
Consumption of Poultry Products	727		
Several times a week		371	51.00
Most days		166	22.80
Once a week		122	16.80
Once every two weeks		37	5.10
Once a month		15	2.10
Never		10	1.40
Once every few months		6	0.80
Education	653		
Bachelor’s Degree		175	26.80
High School Graduate		145	22.20
Some College		145	22.20
Associate’s Degree		70	10.70
Master’s Degree		61	9.30
Trade/Technical/Vocational Training		25	3.80
Professional Degree		14	2.10
Some High School		11	1.70
Doctoral Degree		7	1.10
Income	652		
$35,000–$74,999		222	34.00
$25,000–$34,999		102	15.60
$10,000–$24,999		95	14.50
$75,000–$99,999		82	12.60
$100,000–$149,999		72	11.00
$150,000–$249,999		33	5.10
Less than $10,000		27	4.10
$250,000 or more		10	1.50
Prefer not to respond		10	1.50
Race	652		
White		512	78.50
Black or African American		69	10.60
Hispanic or Latino		36	5.50
Asian		17	2.60
Some other race, ethnicity, or origin		15	2.30
Prefer not to respond		4	0.60
Primary Grocery Shopper	653		
Yes		582	89.10
No		71	10.90
Children Under 18 Living in Home	652		
No		519	79.50
Yes		134	20.50
Number of Children	133		
1		87	65.40
2		33	24.80
3		9	6.80
4		4	3.00

Note: ^a^ Age groups 18–24 and 25–34 were combined because of low representation of each group.

**Table 5:** Descriptive statistics for intentions to adhere to food safety practices for raw chicken and cognitive and affective variables (*N* = 727).

Variable	Video Condition	*M*	*SD*	*Z*	*p*	*r*
Intentions to Adhere to Food Safety Practices (5-point scale)	Control (*n* = 307)	4.75	0.51	15.87	<0.001	0.91
	Expert (*n* = 215)	4.59	0.57	12.88	<0.001	0.88
	SMI (*n* = 205)	4.33	0.82	12.25	<0.001	0.86
Trust in Science (7-point scale)	N/A (pre-video)	5.97	1.03	23.10	<0.001	0.86
Food Safety Knowledge *	N/A (pre-video)	3.80	1.44	12.80	<0.001	0.47
Perceived Threat (7-point scale)	Expert (*n* = 215)	6.64	0.71	13.09	<0.001	0.89
	Control (*n* = 307)	6.33	0.99	15.18	<0.001	0.87
	SMI (*n* = 205)	6.29	1.26	12.33	<0.001	0.86
Perceived Efficacy (7-point scale)	Expert (*n* = 215)	6.63	0.57	12.96	<0.001	0.88
	Control (*n* = 307)	6.63	0.71	15.67	<0.001	0.89
	SMI (*n* = 205)	6.35	1.02	12.47	<0.001	0.87
Trust in Information Source (7-point scale)	Expert (*n* = 215)	6.25	1.06	12.74	<0.001	0.87
	Control (*n* = 307)	5.97	1.28	14.91	<0.001	0.85
	SMI (*n* = 205)	5.00	2.01	8.71	<0.001	0.61
Counterarguing (7-point scale)	SMI (*n* = 205)	4.03	2.15	3.29	0.001	0.23
	Control (*n* = 307)	2.28	1.45	−11.10	<0.001	−0.64
	Expert (*n* = 215)	2.15	1.39	−10.00	<0.001	−0.68

Note: * Indicates the variable mean was obtained by summing the number of correct answers from six questions total. SMI = social media influencer.

**Table 6:** Endpoint rating distribution of trust in information source across food safety video conditions (*N* = 727).

Adjectives—Trust	Endpoint Ratings	Food Safety Video Condition		
		Expert % (*n* = 215)	Control % (*n* = 307)	SMI % (*n* = 205)
Dependable	7 (dependable)	57.67 _a_	47.88 _a_	33.17 _b_
	1 (undependable)	0.93 _a_	2.61 _a_	19.02 _b_
Honest	7 (honest)	60.47 _a_	53.09 _a_	35.61 _b_
	1 (dishonest)	1.86 _a_	1.30 _a_	14.63 _b_
Reliable	7 (reliable)	58.14 _a_	50.81 _a_	34.63 _b_
	1 (unreliable)	1.40 _a_	1.95 _a_	19.51 _b_
Sincere	7 (sincere)	56.74 _a_	50.81 _a_	39.02 _b_
	1 (insincere)	2.33 _a_	2.28 _a_	10.24 _b_
Trustworthy	7 (trustworthy)	60.00 _a_	49.51 _a_	34.63 _b_
	1 (untrustworthy)	1.40 _a_	2.93 _a_	18.52 _b_

Note: Values are column percentages within each condition. Within rows, values sharing a subscript letter do not differ significantly in their column proportions at α = 0.05 confidence level (Bonferroni-adjusted pairwise z tests). SMI = social media influencer.

**Table 7:** Pearson correlations between perceived threat, perceived efficacy, and potential moderator variables with intentions to adhere to food safety practices for handling and preparing raw chicken (*N* = 727).

Variable	1	2	3	4	5	6	7
Intentions (1)	1						
Food Safety Knowledge (2)	0.204 **	1					
Trust in Science (3)	0.134 **	0.195 **	1				
Perceived Risk (4)	0.459 **	0.188 **	0.287 **	1			
Perceived Efficacy (5)	0.496 **	0.157 **	0.262 **	0.588 **	1		
Trust in Information Source (6)	0.282 **	0.048 **	0.195 **	0.218 **	0.277 **	1	
Counterarguing (7)	−0.307 **	−0.220 **	−0.158 **	−0.344 **	−0.358 **	−0.358 **	1

Note: Intentions = Intentions to adhere to food safety practices for handling and preparing raw chicken. ** Correlation is significant at α = 0.01 confidence level.

**Table 8:** Pearson correlations between intentions to adhere to food safety practices for handling and preparing raw chicken and selected sociodemographic characteristics (*n* = 653).

Variable	1	2	3	4	5	6
Intentions (1)	1					
Age (2)	0.459 **	1				
Gender (3)	0.061	−0.039	1			
Education (4)	0.002	−0.061	−0.065	1		
Income (5)	−0.109 **	−0.074	−0.073	0.274	1	
Race (6)	0.038	0.151 **	0.089	−0.005	−0.057	1

Note: Intentions = Intentions to adhere to food safety practices for handling and preparing raw chicken. ** Correlation is significant at α = 0.01 confidence level.

**Table 9:** Confirmatory factor analysis results for respecified measurement model (*N* = 727).

Constructs and Items	Factor Loading	KR-20	CR	AVE
Food Safety Knowledge		0.488	0.624	0.223
Cutting Board Cleaning	0.506			
Risky Turkey Stuffing	0.441			
Thawed Meat Handling	0.602			
Safe Chicken Temp	0.513			
Lowest-Risk Food	0.391			
High-Risk Groups	0.329			
Perceived Threat			0.956	0.783
Item 1	0.860			
Item 2	0.825			
Item 3	0.889			
Item 4	0.916			
Item 5	0.893			
Item 6	0.923			
Perceived Efficacy			0.952	0.768
Item 1	0.846			
Item 2	0.837			
Item 3	0.914			
Item 4	0.847			
Item 5	0.908			
Item 6	0.902			
Trust in Science			0.911	0.631
Item 1	0.802			
Item 2	0.848			
Item 3	0.717			
Item 4	0.883			
Item 5	0.722			
Item 6	0.782			
Trust in Information Source			0.958	0.819
Item 1	0.894			
Item 2	0.912			
Item 3	0.935			
Item 4	0.861			
Item 5	0.921			
Counterarguing			0.951	0.867
Item 1	0.948			
Item 2	0.920			
Item 3	0.925			
Intentions to Adhere to Food Safety Practices			0.595	0.334
Item 1	0.447			
Item 2	0.615			
Item 3	0.651			

**Table 10:** Fit indices of respecified measurement model.

Criteria	Threshold for Acceptable Model Fit	Measurement Model
RMSEA	<0.05–0.08	0.031
SRMR	<0.08	0.036
CFI	>0.90	0.950
TLI	>0.90	0.945

**Table 11:** Model fit of structural model A: effects of information source–message, perceived threat, and perceived efficacy on intentions to adhere to food safety practices.

Criteria	Threshold for Acceptable Model Fit	Measurement Model
RMSEA	<0.05–0.08	0.045
SRMR	<0.08	0.031
CFI	>0.90	0.955
TLI	>0.90	0.945

**Table 12:** Structural model A results: effects of information source–message, perceived threat, and perceived efficacy on intentions to adhere to food safety practices (*N* = 727).

Variable	Source Comparison	Effect Type	Outcome	β	C.I.
Information source–message	Expert vs. SMI	Direct *	Perceived threat	0.344	[0.148, 0.540]
	Expert vs. SMI	Direct *	Perceived efficacy	0.265	[0.122, 0.408]
	Expert vs. SMI	Direct	Intentions	0.084	[−0.013, 0.182]
	Expert vs. control	Direct *	Intentions	−0.211	[−0.408, −0.192]
	SMI vs. control	Direct *	Intentions	−0.300	[0.006, 0.081]
Perceived threat	Expert vs. SMI	Indirect *	Intentions	0.047	[0.004, 0.090]
	Expert vs. control	Indirect *	Intentions	0.048	[0.006, 0.081]
	SMI vs. control	Indirect *	Intentions	−0.004	[−0.032, 0.024]
Perceived efficacy	Expert vs. SMI	Indirect *	Intentions	0.068	[0.018, 0.117]
	Expert vs. control	Indirect	Intentions	0.004	[−0.022, 0.029]
	SMI vs. control	Indirect *	Intentions	−0.070	[−0.110, −0.018]

Note: SMI = social media influencer. Intentions = intentions to adhere to food safety practices for handling and preparing raw chicken. * Indicates significant effects at α = 0.05 confidence level.

**Table 13:** Model fit of structural model B: effects of information source–message, perceived threat, trust in science, food safety knowledge, trust in information source, and counterarguing on intentions to adhere to food safety practices.

Criteria	Threshold for Acceptable Model Fit	Measurement Model
RMSEA	<0.05–0.08	0.038
SRMR	<0.08	0.069
CFI	>0.90	0.955
TLI	>0.90	0.946

**Table 14:** Structural model B results: effects of information source–message, trust in science, food safety knowledge, trust in information source, and counterarguing on intentions to adhere to food safety practices for handling and preparing raw chicken (*N* = 727).

Variable	Source Comparison	Effect Type	Outcome	β
Information source–message	Expert vs. SMI	Direct	Intentions	0.064
	Expert vs. SMI	Direct *	Perceived threat	0.307
	Expert vs. control	Direct *	Intentions	−0.244
	SMI vs. control	Direct *	Intentions	−0.308
Trust in science	N/A	Direct *	Perceived threat	0.364
	Expert vs. SMI	Moderation	Perceived threat	0.030
	Expert vs. SMI	Moderation	Intentions	0.055
	Expert vs. control	Moderation	Intentions	0.022
	SMI vs. control	Moderation	Intentions	−0.032
Food safety knowledge	N/A	Direct *	Intentions	0.178
	Expert vs. SMI	Moderation	Intentions	−0.042
	Expert vs. control	Moderation	Intentions	0.031
	SMI vs. control	Moderation	Intentions	−0.044
Trust in information source	N/A	Direct *	Intentions	0.293
	Expert vs. SMI	Moderation	Intentions	0.029
	Expert vs. control	Moderation	Intentions	−0.019
	SMI vs. control	Moderation	Intentions	−0.115
Counterarguing	N/A	Direct	Intentions	0.046
	Expert vs. SMI	Moderation	Intentions	−0.047
	Expert vs. control	Moderation	Intentions	0.019
	SMI vs. control	Moderation	Intentions	−0.109

Note: SMI = social media influencer. Intentions = intentions to adhere to food safety practices for handling and preparing raw chicken. * Indicates significant effects at α = 0.05 confidence level.

**Table 15:** Model 1: Main effects of sociodemographic variables on intentions to adhere to food safety practices for handling and preparing raw chicken (*n* = 653).

Main Effects	β	SE	*t*	*p*	95% C.I.
Information Source–Message	-	-	-	-	-
SMI *	−0.277	0.064	−6.218	0.000	[−0.525, −0.273]
Expert *	−0.095	0.064	−2.094	0.037	[−0.262, −0.008]
Age	-	-	-	-	-
18–35	0.023	0.087	0.528	0.598	[−0.126, 0.218]
Gender	-	-	-	-	-
Male	0.055	0.055	1.324	0.186	[−0.035, 0.180]
Race	-	-	-	-	-
Black or African American	−0.022	0.093	−0.536	0.592	[−0.232, 0.133]
Hispanic or Latino *	−0.087	0.125	−2.047	0.041	[−0.501, −0.010]
Asian and Other	0.018	0.124	0.431	0.666	[−0.191, 0.298]
Income	-	-	-	-	-
$24,999 or less	−0.008	0.076	−0.171	0.864	[−0.161, 0.135]
$25,000–$34,999	0.014	0.040	0.301	0.764	[−0.066, 0.090]
$75,000 or more	−0.069	0.017	−1.381	0.168	[−0.058, 0.010]
Education	-	-	-	-	-
High School or Less	−0.037	0.063	−0.879	0.380	[−0.178, 0.068]
Masters, Doctoral, and Professional Degree	0.018	0.088	0.404	0.686	[−0.138, 0.209]

Note: *R*^2^ = 0.080. Adjusted *R*^2^ = 0.061. SMI = social media influencer. * Indicate significant effects at α = 0.05 confidence level.

**Table 16:** Model 2: Main effects and interaction effects of sociodemographic variables on intentions to adhere to food safety practices for handling and preparing raw chicken (*n* = 653).

	β	SE	*t*	*p*	95% C.I.
**Main Effects**					
Information Source–Message	-	-	-	-	-
SMI *	−0.292	0.158	−2.659	0.008	[−0.731, −0.110]
Expert	0.022	0.149	0.205	0.837	[−0.262, 0.323]
Age	-	-	-	-	-
18–35	0.053	0.114	0.943	0.346	[−0.116, 0.331]
Gender	-	-	-	-	-
Male *	0.168	0.087	2.545	0.011	[0.050, 0.391]
Race	-	-	-	-	-
Black or African American	0.052	0.153	0.761	0.447	[−0.184, 0.418]
Hispanic or Latino *	−0.066	0.177	−1.084	0.279	[−0.540, 0.156]
Asian and Other	0.055	0.153	1.085	0.279	[−0.135, 0.468]
Income	-	-	-	-	-
$24,999 or less	−0.076	0.115	−1.073	0.284	[−0.349, 0.102]
$25,000–$34,999	−0.026	0.061	−0.367	0.714	[−0.143, 0.098]
$75,000 or more	−0.114	0.027	−1.462	0.144	[−0.093, 0.014]
Education	-	-	-	-	-
High School or Less	−0.072	0.098	−1.092	0.275	[−0.301, 0.086]
Masters, Doctoral, and Professional Degree	−0.028	0.146	−0.384	0.701	[−0.343, 0.231]
**Interaction Effects**					
Information Source–Message x Age	-	-	-	-	-
Expert × Age 18–35	−0.004	0.497	−0.089	0.929	[−1.020, 0.932]
SMI × Age 18–35	−0.051	0.216	−0.807	0.420	[−0.597, 0.249]
Information Source–Message x Gender	-	-	-	-	-
Expert × Male	−0.147	0.134	−1.899	0.058	[−0.519, 0.009]
SMI × Male	−0.144	0.136	−1.796	0.073	[−0.510, 0.023]
Information Source–Message x Race	-	-	-	-	-
Expert × Black or African American	−0.001	0.227	−0.018	0.986	[−0.450, 0.442]
Expert × Hispanic or Latino	0.000	0.277	0.006	0.995	[−0.543, 0.547]
Expert × Asian and Other	0.002	0.348	0.039	0.969	[−0.671, 0.698]
SMI × Black or African American	−0.108	0.244	−1.744	0.082	[−0.905, 0.054]
SMI × Hispanic or Latino	0.016	0.493	0.368	0.713	[−0.787, 1.150]
SMI × Asian and Other	−0.070	0.412	−1.546	0.123	[−1.445, 0.172]
Information Source–Message x Income	-	-	-	-	-
Expert × $24,999 or Less	0.013	0.194	0.223	0.824	[−0.338, 0.424]
Expert × $25,000–$34,999	−0.008	0.096	−0.125	0.900	[−0.200, 0.176]
Expert × $75,000 or More	−0.068	0.043	−0.822	0.412	[−0.121, 0.050]
SMI × $24,999 or Less	0.116	0.181	1.637	0.102	[−0.059, 0.652]
SMI × $25,000–$34,999	0.086	0.102	1.281	0.201	[−0.070, 0.331]
SMI × $75,000 or More	0.144	0.045	1.880	0.061	[−0.004, 0.172]
Information Source–Message x Education	-	-	-	-	-
Expert × High School or Less	0.020	0.153	0.320	0.749	[−0.252, 0.350]
Expert × Masters, Doctoral, and Professional Degree	0.061	0.216	0.917	0.359	[−0.226, 0.622]
SMI × High School or Less	0.026	0.154	0.425	0.671	[−0.237, 0.369]
SMI × Masters, Doctoral, and Professional Degree	0.056	0.221	0.945	0.345	[−0.225, 0.643]

Note: Change in *R*^2^ = 0.116. Adjusted *R*^2^ = 0.065. SMI = social media influencer. * Indicates significant effects at α = 0.05 confidence level.

**Table 17:** Conclusions for hypotheses.

No.	RQ	Hypothesis	Supported
H1	1	Participants who watched a food safety video about raw chicken from an expert source who provided factual information reported more intentions to adhere to food safety practices for raw chicken than those who watched a social media influencer video who provided misinformation.	No
H2	2	Participants who watched a food safety video about raw chicken from an expert source who provided factual information reported stronger perceived threat than those who watched a social media influencer who provided misinformation.	Yes
H3	2	Perceived threat contributed to an indirect pathway from information source–message and intentions to adhere to food safety practices for raw chicken.	Yes
H4	2	Participants who watched a food safety video about raw chicken from an expert source who provided factual information reported stronger perceived efficacy than those who watched a social media influencer video who provided misinformation.	Yes
H5	2	Perceived efficacy contributed to an indirect pathway from information source–message and intentions to adhere to food safety practices for raw chicken.	Yes
H6	3	When participants with more trust in science watched an expert source food safety video about raw chicken who provided factual information, they reported stronger perceived threat than participants who watched a social media influencer video who provided misinformation.	No
H7	3	Trust in science moderated the effect of information source–message on intentions to adhere to food safety practices for raw chicken through perceived threat.	No
H8	4	Food safety knowledge moderated the effect of information source–message on intentions to adhere to food safety practices for raw chicken.	No
H9	4	Trust in information source moderated the effect of information source–message on intentions to adhere to food safety practices for raw chicken.	No
H10	4	Participants who watched a food safety video about raw chicken from a social media influencer source who provided misinformation reported more counterarguing than participants who watched an expert video who provided factual information.	Yes
H11	4	Counterarguing moderated the effect of information source–message on intentions to adhere to food safety practices for raw chicken.	No
H12	4	Sociodemographic factors (i.e., age, gender, education, income, and race) moderated the effect of information source–message on intentions to adhere to food safety practices for raw chicken.	No

## Data Availability

The data presented in this study are available on request from the corresponding author due to privacy and confidentiality restrictions.

## Supplementary Materials

The following supporting information can be downloaded at: https://www.mdpi.com/article/10.3390/foods15183170/s1, Figure S1: Structural Model A; Figure S2: Structural Model B.

## References

[1] U.S. Food Safety and Inspection Service. Foodborne Illness and Disease. http://www.fsis.usda.gov/food-safety/foodborne-illness-and-disease.

[2] Holst M.M. (2025). Contributing Factors of Foodborne Illness Outbreaks—National Outbreak Reporting System, United States, 2014–2022. MMWR Surveill. Summ..

[3] Henderson B. USDA-FSIS Work Plans for 2025 Prioritize Regulating Salmonella in Poultry. https://www.food-safety.com/articles/10055-usda-fsis-work-plans-for-2025-prioritize-regulating-salmonella-in-poultry.

[4] Centers for Disease Control and Prevention Chicken and Food Poisoning. https://www.cdc.gov/food-safety/foods/chicken.html.

[5] U.S. Food Safety and Inspection Service Poultry. http://www.fsis.usda.gov/food-safety/safe-food-handling-and-preparation/poultry.

[6] Beach C. (2023). What’s Old Is New Again This New Year. Food Safety News.

[7] Goldberg A. (2025). Why TikTok Influencers Are Eating Raw Beef (and Why You Shouldn’t). Yahoo Lifestyle.

[8] Cost B. (2022). I Eat Raw Chicken—But I’m Healthier and More Sensual than Ever. New York Post.

[9] Godwin N. (2023). Traditional vs New Media: An Examination of News Consumption Patterns amongst Media Users. World J. Adv. Res. Rev..

[10] Ma J., Almanza B., Ghiselli R., Vorvoreanu M., Sydnor S. (2017). Food Safety Information on the Internet: Consumer Media Preferences. Food Prot. Trends.

[11] Centers for Disease Control and Prevention Preventing Food Poisoning. https://www.cdc.gov/food-safety/prevention/index.html.

[12] Bode L., Vraga E.K. (2015). In Related News, That Was Wrong: The Correction of Misinformation Through Related Stories Functionality in Social Media. J. Commun..

[13] Hardoš P. (2018). Who Exactly Is an Expert? On the Problem of Defining and Recognizing Expertise. Sociológia-Slovak. Sociol. Rev..

[14] Barta S., Belanche D., Fernández A., Flavián M. (2023). Influencer Marketing on TikTok: The Effectiveness of Humor and Followers’ Hedonic Experience. J. Retail. Consum. Serv..

[15] Chaffey D., Ellis-Chadwick F. (2019). Digital Marketing.

[16] Ozdemir-Guzel S., Bas Y.N., Stylos N., Rahimi R., Okumus B., Williams S. (2021). Gen Z Tourists and Smart Devices. Generation Z Marketing and Management in Tourism and Hospitality. The Future of the Industry.

[17] Chambers A.V., Baker M.T., Leggette H.R., Osburn W.N., Lu P. (2023). Effects of Message Framing and Information Source on Consumers’ Attitudes toward an Amino Acid-Based Alternative Meat Curing System. Foods.

[18] Chon M.-G., Xu L., Kim J., Liu J. (2025). Understanding Active Communicators on the Food Safety Issue: Conspiratorial Thinking, Organizational Trust, and Communicative Actions of Publics in China. Am. Behav. Sci..

[19] Shan L., Jiao X., Wu L., Shao Y., Xu L. (2022). Influence of Framing Effect on Consumers’ Purchase Intention of Artificial Meat—Based on Empirical Analysis of Consumers in Seven Cities. Front. Psychol..

[20] Kalani D.V.A. (2023). An Overview of Information Sources. Int. J. Res. Soc. Sci. Mod. Lang..

[21] Yang C.-X., Baker L.M. (2024). Impact of Reliable News Information on Consumers’ Perceptions and Information Seeking Intentions from a Food Safety Risk. Br. Food J..

[22] Thomas M.S., Feng Y. (2021). Consumer Risk Perception and Trusted Sources of Food Safety Information during the COVID-19 Pandemic. Food Control.

[23] Zhang A.L., Lu H. (2023). Scientists as Influencers: The Role of Source Identity, Self-Disclosure, and Anti-Intellectualism in Science Communication on Social Media. Soc. Media + Soc..

[24] Rembischevski P., Caldas E.D. (2023). Consumers’ Trust in Different Sources of Information Related to Food Hazards and Their Judgment of Government Performance—A Cross-Sectional Study in Brazil. Foods.

[25] Settle Q., Harvey L., Ruth T., Rumble J.N. (2023). Young Mothers’ Trust of Celebrities and Influencers for Food Safety and Nutrition Information. J. Appl. Commun..

[26] Witte K. (1992). Putting the Fear Back into Fear Appeals: The Extended Parallel Process Model. Commun. Monogr..

[27] Zou W., Zhang W.J., Tang L. (2020). What Do Social Media Influencers Say about Health? A Theory-Driven Content Analysis of Top Ten Health Influencers’ Posts on Sina Weibo. J. Health Commun..

[28] Liao C., Luo Y., Zhu W. (2020). Food Safety Trust, Risk Perception, and Consumers’ Response to Company Trust Repair Actions in Food Recall Crises. Int. J. Environ. Res. Public Health.

[29] Zhu Y., Wen X., Chu M., Zhang G., Liu X. (2021). Consumers’ Food Safety Risk Communication on Social Media Following the Suan Tang Zi Accident: An Extended Protection Motivation Theory Perspective. Int. J. Environ. Res. Public Health.

[30] Chou F.S., Wang C.C., Lai M.C., Tung C.H., Yang Y.J., Tsai K.H. (2020). Persuasiveness of Organic Agricultural Products: Argument Strength, Health Consciousness, Self-Reference, Health Risk, and Perceived Fear. Br. Food J..

[31] Manning L., Grant J.H. (2024). Food Safety Management Systems: Determining What Is ‘Safe Enough’. Trends Food Sci. Technol..

[32] Zhang X., Zhou S. (2019). Clicking Health Risk Messages on Social Media: Moderated Mediation Paths through Perceived Threat, Perceived Efficacy, and Fear Arousal. Health Commun..

[33] Liu-Lastres B., Wen H. (2023). Using the Extended Parallel Process Model (EPPM) to Explore US Consumers’ Dining Behaviors during COVID-19. Br. Food J..

[34] Baba F.V., Esfandiari Z. (2023). Theoretical and Practical Aspects of Risk Communication in Food Safety: A Review Study. Heliyon.

[35] Shumaker E.T., Kirchner M., Cates S.C., Shelley L., Goulter R., Goodson L., Bernstein C., Lavallee A., Jaykus L.-A., Chapman B. (2022). Observational Study of the Impact of a Food Safety Intervention on Consumer Poultry Washing. J. Food Prot..

[36] Rui J.R., Yang K., Chen J. (2021). Information Sources, Risk Perception, and Efficacy Appraisal’s Prediction of Engagement in Protective Behaviors against COVID-19 in China: Repeated Cross-Sectional Survey. JMIR Hum. Factors.

[37] Man S.-S., Wen H., Zhao L., So B.C.-L. (2023). Role of Trust, Risk Perception, and Perceived Benefit in COVID-19 Vaccination Intention of the Public. Healthcare.

[38] Byrd K., Her E., Fan A., Liu Y., Leitch S. (2022). Consumers’ Threat and Coping Appraisals of in-Restaurant Dining during a Pandemic—The Moderating Roles of Conflicting Information and Trust-in-Science and Scientists. Int. J. Hosp. Manag..

[39] Entradas M. (2022). In Science We Trust: The Effects of Information Sources on COVID-19 Risk Perceptions. Health Commun..

[40] Wingen T., Posten A.C., Dohle S. (2025). No Evidence for Causal Effects of Trust in Science on Intentions for Health-Related Behavior. Commun. Psychol..

[41] Kito Y. (2025). What Drives Risky Eating Behavior: Applying an Extended Theory of Planned Behavior to Raw or Undercooked Chicken Consumption in Japan. Food Res. Int..

[42] Food and Agriculture Organization of the United Nations (2025). Food Safety Is Trust: FAO Calls for Science as Shared Language. https://www.fao.org/food-safety/news/detail/Food-Safety-is-Trust-FAO-Calls-for-Science-as-Shared-Language/en.

[43] Begum M., Alam M.J., Parikh P., De Steur H. (2025). Understanding Food Safety Knowledge, Attitude, and Practices of Consumers and Vendors: An Umbrella Review. Food Control.

[44] Melios S., Asimakopoulou A.A., Greene C.M., Crofton E., Grasso S. (2025). Food-Related Fake News, Misleading Information, Established Misconceptions, and Food Choice. Curr. Opin. Food Sci..

[45] Soon-Sinclair J.M., Ha T.M., Limon M.R., Vanany I., Ongkunaruk P., Voe P., Dao C.D. (2024). Consumers’ Raw Poultry Washing Practices: A Cross-Sectional and Observational Study in Eight Southeast Asian Countries. Food Control.

[46] Katiyo W., Kock H.L., Coorey R., Buys E.M. (2019). Assessment of Safety Risks Associated with Handling Chicken as Based on Practices and Knowledge of a Group of South African Consumers. Food Control.

[47] Ballout R., Toufeili I., Kharroubi S.A., Kassem I.I. (2024). Raw Meat Consumption and Food Safety Challenges: A Survey of Knowledge, Attitudes, and Practices of Consumers in Lebanon. Foods.

[48] Ishra R., Sharif S., Soar J., Khanam R. (2026). Role of Food Safety Risk Perception and Attitude on Knowledge and Practice of Adult Consumers in Bangladesh: A Serial Mediation Model. Food Sci. Nutr..

[49] Alzahrani S.H. (2024). The Impact of Health Beliefs and Trust in Health Information Sources on SARS-CoV-2 Vaccine Uptake. Front. Public Health.

[50] Wilkins E.J., Miller H.M., Tilak E., Schuster R.M. (2018). Communicating Information on Nature-Related Topics: Preferred Information Channels and Trust in Sources. Environ. Commun..

[51] Hovland C., Janis I., Kelley H. (1953). Communication and Persuasion.

[52] Kaňková J., Binder A., Matthes J. (2024). Helpful or Harmful? Navigating the Impact of Social Media Influencers’ Health Advice: Insights from Health Expert Content Creators. BMC Public Health.

[53] Lee D.N., Liu J., Stevens H., Oduguwa K., Stevens E.M. (2024). Does Source Matter? Examining the Effects of Health Experts, Friends, and Social Media Influencers on Young Adult Perceptions of Instagram e-Cigarette Education Messages. Drug Alcohol. Depend..

[54] Liu X., Zheng X. (2024). The Persuasive Power of Social Media Influencers in Brand Credibility and Purchase Intention. Humanit. Soc. Sci. Commun..

[55] Chen J., Wang Y. (2021). Social Media Use for Health Purposes: Systematic Review. J. Med. Internet Res..

[56] Zhou S., Shapiro M.A. (2020). Effects of Egocentric Projection and Identification on Narrative Persuasion in Foodborne Illness Messages. J. Health Commun..

[57] Walter N., Cohen J. (2019). When Less Is More and More Is Less: The Paradoxical Metacognitive Effects of Counterarguing. Commun. Monogr..

[58] Feng G.C., Luo Y., Yu Z., Wen J. (2023). Effects of Rhetorical Devices on Audience Responses with Online Videos: An Augmented Elaboration Likelihood Model. PLoS ONE.

[59] Dudley M.Z., Squires G.K., Petroske T.M., Dawson S., Brewer J. (2023). The Use of Narrative in Science and Health Communication: A Scoping Review. Patient Educ. Couns..

[60] Richter S., Richter A., Franklin D. (2026). Sticky Narratives: How Micro-Influencers Engage and Retain Their Audience Through Storytelling. Australas. Mark. J..

[61] Roberts K. (2020). Self-Reported Food Safety Behaviors in Independent Chinese and Mexican Restaurants in Kansas. Food Prot. Trends.

[62] Carstens C.K., Salazar J.K., Sharma S.V., Chan W., Darkoh C. (2022). Food Safety Attitudes, Behaviors, and Hygiene Measures among Predominantly Low-Income Parents in Houston, Texas. J. Food Prot..

[63] Leung W.-T., Li S., Yu P.H.-F., Chan S.-W. (2025). An Assessment of Sociodemographic Factors, Attitude, Knowledge, and Practices of the Elderly’s Caregivers with Respect to Elderly Food Safety. Foods.

[64] Miftari I., Imami D., Kaliji S.A., Canavari M., Gjokaj E. (2024). Analyzing Consumer Perceptions about Food Safety by Applying the Food-Related Lifestyle Approach. Ital. J. Food Saf..

[65] Yi N.-Y., Choi J.-H. (2025). Age Differences in Food Safety Knowledge, Attitudes, and Practices among Consumers in Seoul. Nutr. Res. Pract..

[66] Zanetta L.D., Mucinhato R.M.D., Hakim M.P., Stedefeldt E., da Cunha D.T. (2022). What Motivates Consumer Food Safety Perceptions and Beliefs? A Scoping Review in BRICS Countries. Foods.

[67] Vatral C.D., Gilman A.D., Quinlan J.J. (2022). Consumer Awareness of the Message Not To Wash Raw Poultry, Current Practices, and Barriers to Following That Message. J. Food Prot..

[68] Kosa K.M., Cates S.C., Godwin S., Chambers E. (2017). Barriers to Using a Food Thermometer When Cooking Poultry at Home: Results from a National Survey. Food Prot. Trends.

[69] Chandrasekaran R., Sadiq T M., Moustakas E. (2024). Racial and Demographic Disparities in Susceptibility to Health Misinformation on Social Media: National Survey-Based Analysis. J. Med. Internet Res..

[70] Rivera Y.M., Corpuz K., Karver T.S. (2025). Engagement With and Use of Health Information on Social Media Among US Latino Individuals: National Cross-Sectional Survey Study. J. Med. Internet Res..

[71] Başkaya Sezer D. (2024). Socio-Demographic Determinants of Good Food Safety Practices for Young, Educated Food Handlers in Türkiye. Food Health.

[72] Benitez J.A. (2026). Risky Reels: Information Source Effect on Consumers’ Intentions to Adhere to Food Safety Practices for Raw Chicken, Collard Greens, and Raw Milk. Doctoral Dissertation.

[73] Campbell D.T., Stanley J.C. (1963). Experimental and Quasi-Experimental Designs for Research.

[74] Montoya A.K. (2022). Selecting a Within- or between-Subject Design for Mediation: Validity, Causality, and Statistical Power. Multivar. Behav. Res..

[75] Lam C., Wolfe J. (2023). An Introduction to Quasi-Experimental Research for Technical and Professional Communication Instructors. J. Bus. Tech. Commun..

[76] Habibi S.A., Salim L. (2021). Static vs. Dynamic Methods of Delivery for Science Communication: A Critical Analysis of User Engagement with Science on Social Media. PLoS ONE.

[77] Montes M., Wargo J., Jones-Jang S.M., Quan S., Lai B., Riobueno-Naylor A. (2025). Evaluating Video-Based Science Communications Practices: A Systematic Review. J. Sci. Commun..

[78] Young D.G., Jamieson K.H., Poulsen S., Goldring A. (2018). Fact-Checking Effectiveness as a Function of Format and Tone: Evaluating FactCheck.Org and FlackCheck.Org. Journal. Mass. Commun. Q..

[79] HeyGen (2025). [AI Video Generation Platform]. https://www.heygen.com/.

[80] Leiker D., Gyllen A.R., Eldesouky I., Cukurova M. (2023). Generative AI for Learning: Investigating the Potential of Learning Videos with Synthetic Virtual Instructors. Proceedings of the International Conference on Artificial Intelligence in Education.

[81] Netland T., Dzengelevski O., Tesch K., Kwasnitschka D. (2025). Comparing Human-Made and AI-Generated Teaching Videos: An Experimental Study on Learning Effects. Comput. Educ..

[82] Shu J., Kou H., Zhang J., Zhang T., Zhang H., Xu T., Zhou Y. (2025). AI-Generated Instructional Videos: A Systematic Review of Learning Impacts, Applications, and Research Perspectives. Proceedings of the 2025 7th International Conference on Computer Science and Technologies in Education (CSTE).

[83] Costello L., Rutherford T. (2019). Expert? What Does That Mean? Describing the Term “Expert” in Agricultural Communications, Education, Extension, and Leadership Research. J. Appl. Commun..

[84] Powell J., Pring T. (2024). The Impact of Social Media Influencers on Health Outcomes: Systematic Review. Soc. Sci. Med..

[85] AlDahoul N., Rahwan T., Zaki Y. (2025). AI-Generated Faces Influence Gender Stereotypes and Racial Homogenization. Sci. Rep..

[86] U.S. Census Bureau (2021). U.S. Population More Racially, Ethnically Diverse than in 2021.

[87] Eom D., Molder A.L., Tosteson H.A., Howell E.L., DeSalazar M., Kirschner E., Scheufele D.A. (2025). Race and Gender Biases Persist in Public Perceptions of Scientists’ Credibility. Sci. Rep..

[88] Greve-Poulsen K., Larsen F.K., Pedersen R.T., Albæk E. (2023). No Gender Bias in Audience Perceptions of Male and Female Experts in the News: Equally Competent and Persuasive. Int. J. Press/Politics.

[89] Haut K., Wohn C., Antony V., Goldfarb A., Welsh M., Sumanthiran D., Jang J., Ali M.R., Hoque E. (2021). Could You Become More Credible by Being White? Assessing Impact of Race on Credibility with Deepfakes. arXiv.

[90] Liu T., Wang P., Pan D., Liu R. (2025). Credibility of AI Generated and Human Video Doctors and the Relationship to Social Media Use. Front. Public Health.

[91] Audio Extractor: Extract Audio from Video—Online, Free. https://restream.io/tools/audio-extractor.

[92] O’Neill R. (2025). Rethinking the ‘Wellness Influencer’: Medical Doctors, Lifestyle Expertise and the Question of Credentials. Int. J. Cult. Stud..

[93] Byrd-Bredbenner C., Wheatley V., Schaffner D., Bruhn C., Blalock L., Maurer J. (2007). Development and Implementation of a Food Safety Knowledge Instrument. J. Food Sci. Educ..

[94] Rumble J.N., Wu Y.L., Tully K., Ruth T.K., Ellis J.D., Lamm A.J. (2020). A Mixed-Methods Comparison of Self-Reported and Conversational Trust in Science. J. Appl. Commun..

[95] National Science Foundation 2018 Indicators Report: Science and Technology: Public Attitudes and Understanding. https://www.nsf.gov/statistics/2018/nsb20181/report/sections/science-and-technology-public-attitudes-and-understanding/public-attitudes-about-s-t-in-general.

[96] Witte K. (1996). Predicting Risk Behaviors: Development and Validation of a Diagnostic Scale. J. Health Commun..

[97] Shi J., Smith S.W. (2016). The Effects of Fear Appeal Message Repetition on Perceived Threat, Perceived Efficacy, and Behavioral Intention in the Extended Parallel Process Model. Health Commun..

[98] Ohanian R. (1990). Construction and Validation of a Scale to Measure Celebrity 135 Endorsers’ Perceived Expertise, Trustworthiness, and Attractiveness. J. Advert..

[99] Yan X. (2022). Being Targeted: How Counter-Arguing and Message Relevance Mediate the Effects of Cultural Value Appeals on Disease Prevention Attitudes and Behaviors. Front. Psychol..

[100] Silvia P.J. (2006). Reactance and the Dynamics of Disagreement: Multiple Paths from Threatened Freedom to Resistance to Persuasion. Eur. J. Soc. Psych..

[101] Madjdian D.S., Asseldonk M., Talsma E.F., Dione M., Ilboudo G., Roesel K., Vet E. (2024). Empowering Consumers to Purchase Safe Ready-to-Eat Chicken from Street Restaurants in Ouagadougou, Burkina Faso: Impact of a Multi-Media Behavior Change Campaign. Sci. Rep..

[102] Hughes J.L., Camden A.A., Yangchen T. (2016). Rethinking and Updating Demographic Questions: Guidance to Improve Descriptions of Research Samples. Psi Chi J. Psychol. Res..

[103] Haynes S.N., Richard D.C.S., Kubany E.S. (1995). Content Validity in Psychological Assessment: A Functional Approach to Concepts and Methods. Psychol. Assess..

[104] Wolf E.J., Harrington K.M., Clark S.L., Miller M.W. (2013). Sample Size Requirements for Structural Equation Models: An Evaluation of Power, Bias, and Solution Propriety. Educ. Psychol. Meas..

[105] McDonald R.P. (1999). Test Theory: A Unified Treatment.

[106] Hox J.J. (2021). Confirmatory Factor Analysis. The Encyclopedia of Research Methods in Criminology and Criminal Justice.

[107] Anselmi P., Colledani D., Robusto E. (2019). A Comparison of Classical and Modern Measures of Internal Consistency. Front. Psychol..

[108] Ekolu S.O., Quainoo H. (2019). Reliability of Assessments in Engineering Education Using Cronbach’s Alpha, KR and Split-Half Methods. Glob. J. Eng. Educ..

[109] McCrae R.R., Kurtz J.E., Yamagata S., Terracciano A. (2011). Internal Consistency, Retest Reliability, and Their Implications for Personality Scale Validity. Personal. Soc. Psychol. Rev..

[110] Ntumi S., Agbenyo S., Bulala T. (2023). Estimating the Psychometric Properties (“Item Difficulty, Discrimination and Reliability Indices”) of Test Items Using the Kuder-Richardson Approach (KR-20). Shanlax Int. J. Educ..

[111] Borda D., Mihalache O.A., Dumitraşcu L., Gafițianu D., Nicolau A.I. (2021). Romanian Consumers’ Food Safety Knowledge, Knowledge on Certified Labelled Food and Trust in Information Sources. Food Control.

[112] Cohen J. (1988). Statistical Power Analysis for the Behavioral Sciences.

[113] Buchberger E.S., Ngo C.T., Peikert A., Brandmaier A.M., Werkle-Bergner M. (2024). Estimating Statistical Power for Structuralequation Models in Developmental Cognitive Science: A Tutorial in R. Behav. Res..

[114] Memon M.A., Ting H., Cheah J.H., Thurasamy R., Chuah F., Cham T.H. (2020). Sample Size for Survey Research: Review and Recommendations. J. Appl. Struct. Equ. Model..

[115] Kline R.B. (2016). Principles and Practice of Structural Equation Modeling.

[116] Olinsky A., Chen S., Harlow L. (2003). The Comparative Efficacy of Imputation Methods for Missing Data in Structural Equation Modeling. Eur. J. Oper. Res..

[117] Hair J.F., Black W.C., Babin B.J., Anderson R.E. (2010). Multivariate Data Analysis: A Global Perspective.

[118] Kim H.-Y. (2019). Statistical Notes for Clinical Researchers: Simple Linear Regression 3—Residual Analysis. Restor. Dent. Endod..

[119] Pavlov G., Maydeu-Olivares A., Shi D. (2021). Using the Standardized Root Mean Squared Residual (SRMR) to Assess Exact Fit in Structural Equation Models. Educ. Psychol. Meas..

[120] Xia Y., Yang Y. (2019). RMSEA, CFI, and TLI in Structural Equation Modeling with Ordered Categorical Data: The Story They Tell Depends on the Estimation Methods. Behav. Res. Methods.

[121] Flannelly K.J., Flannelly L.T., Jankowski K.R.B. (2018). Threats to the Internal Validity of Experimental and Quasi-Experimental Research in Healthcare. J. Health Care Chaplain..

[122] Czapla J.J., Laursen L.N. How to Design Quasi-Experiments in Organizations? A Systematic Review of Innovation Research. Proceedings of the ISPIM Conference Proceedings.

[123] Moseley A.M., Pinheiro M.B. (2022). Research Note: Evaluating Risk of Bias in Randomised Controlled Trials. J. Physiother..

[124] Maciejewski M.L. (2018). Quasi-experimental design. Biostat. Epidemiol..

[125] Fornell C., Larcker D.F. (1981). Evaluating Structural Equation Models with Unobservable Variables and Measurement Error. J. Mark. Res..

[126] Parrella J.A., Leggette H.R., Lu P., Wingenbach G., Baker M., Murano E. (2023). Evaluating Factors Explaining U.S. Consumers’ Behavioral Intentions toward Irradiated Ground Beef. Foods.

[127] Comrey A.L. (1992). A First Course in Factor Analysis.

[128] Boateng G.O., Neilands T.B., Frongillo E.A., Melgar-Quiñonez H.R., Young S.L. (2018). Best Practices for Developing and Validating Scales for Health, Social, and Behavioral Research: A Primer. Front. Public Health.

[129] Shi D., Lee T., Maydeu-Olivares A. (2019). Understanding the Model Size Effect on SEM Fit Indices. Educ. Psychol. Meas..

[130] Bentler P.M. (1990). Comparative Fit Indexes in Structural Models. Psychol. Bull..

[131] Steiger J.H. (1990). Structural Model Evaluation and Modification: An Interval Estimation Approach. Multivar. Behav. Res..

